# Mixed Hypertrophic and Dilated Phenotype of Cardiomyopathy in a Patient With Homozygous In-Frame Deletion in the *MyBPC3* Gene Treated as Myocarditis for a Long Time

**DOI:** 10.3389/fphar.2020.579450

**Published:** 2020-09-25

**Authors:** Olga Blagova, Indira Alieva, Eugenia Kogan, Alexander Zaytsev, Vsevolod Sedov, S. Chernyavskiy, Yulia Surikova, Ilya Kotov, Elena V. Zaklyazminskaya

**Affiliations:** ^1^Sechenov First Moscow State Medical University, Sechenov University, Moscow, Russia; ^2^Medical Genetics Laboratory, Petrovsky National Research Centre of Surgery, Moscow, Russia; ^3^Department of Bioinformatics, Centre of Genetics and Reproductive Medicine “Genetico”, Moscow, Russia

**Keywords:** hypertrophic cardiomyopathy, dilated cardiomyopathy, *MyBPC3* gene, heart failure progression, bi-allelic mutations, myocarditis, endomyocardial biopsy

## Abstract

Hypertrophic cardiomyopathy (HCM) is the most common inherited disease, with a prevalence of 1:200 worldwide. The cause of HCM usually presents with an autosomal dominant mutation in the genes encoding one of more than 20 sarcomeric proteins, incomplete penetrance, and variable expressivity. HCM classically manifests as an unexplained thickness of the interventricular septum (IVS) and left ventricular (LV) walls, with or without the obstruction of the LV outflow tract (LVOT), and variable cardiac arrhythmias. Here, we present a rare case of mixed cardiomyopathy (cardiac hypertrophy and dilation) and erythrocytosis in a young patient. A 27-year-old man was admitted to the clinic due to biventricular heart failure (HF) NYHA class III. Personal medical records included a diagnosis of dilated cardiomyopathy (DCM) since the age of 4 years and were, at the time, considered an outcome of myocarditis. Severe respiratory infection led to circulatory decompensation and acute femoral thrombosis. The combination of non-obstructive LV hypertrophy (LV walls up to 15 mm), LV dilatation, decreased contractility (LV EF 24%), and LV apical thrombosis were seen. Cardiac MRI showed a complex pattern of late gadolinium enhancement (LGE). Endomyocardial biopsy (EMB) revealed primary cardiomyopathy with intravascular coagulation and an inflammatory response. No viral genome was detected in the plasma or EMB samples. Whole exome sequencing (WES) revealed a homozygous in-frame deletion p.2711_2737del in the *MyBPC3* gene. The clinically unaffected mother was a heterozygous carrier of this deletion, and the father was unavailable for clinical and genetic testing. Essential erythrocytosis remains unexplained. No significant improvement was achieved by conventional treatment, including prednisolone 40 mg therapy. ICD was implanted due to sustained VT and high risk of SCD. Orthotopic heart transplantation (HTx) was considered optimal. Early manifestation combined hypertrophic and dilated phenotype, and progression may reflect a complex genotype with more than one pathogenic allele and/or a combination of genetic diseases in one patient.

## Introduction

Primary myocardial diseases are one of the most difficult to diagnose and treat in cardiology. The most common inherited disease of the myocardium is hypertrophic cardiomyopathy (HCM), with an overall prevalence of approximately 1:200 worldwide (Maron et al., 2018). The disease is characterized by cardiac hypertrophy unexplained by pressure or volume overload, non-dilated left ventricle, and preserved or increased ejection fraction (Marian and Braunwald, 2017). HCM classically manifests as an unexplained thickness of the interventricular septum (IVS) and left ventricular (LV) walls with or without obstruction of the left ventricular outflow tract (LVOT), and variable arrhythmias (Marian and Braunwald, 2017). The cause of HCM is usually an autosomal dominant mutation in the genes encoding one of more than 20 sarcomeric proteins, incomplete penetrance, and variable expressivity (Kuusisto, 2020). The most common findings are causative variants of the *MYH7* and *MyBPC3* genes (Marian and Braunwald, 2017; Maron et al., 2018; Kuusisto, 2020). Generally, 5% to 7% of HCM index cases have two or more presumably damaging variants in the genes of interest; such patients have earlier manifestations, more pronounced cardiac hypertrophy, higher risk of sudden cardiac death, and increased risk of heart failure (Wang et al., 2014; Marian and Braunwald, 2017).

Multi-systemic disorders, which can mimic “sarcomeric” HCM and progress to heart failure, account for up to 40% of all unexplained myocardial hypertrophy (Authors/Task Force members et al., 2014). These phenocopies have rather different genetic origins, molecular pathogenesis, and natural course of disease. Precise etiological diagnosis is significant because the short-term and long-term prognosis may widely differ, and some of these phenocopies have gene-specific targets available for therapy (Fabry disease, TTR-amyloidosis, etc.). Various epigenetic factors and mechanisms may also significantly modulate the clinical course of the disease and prognosis; an important factor being inflammation. The driving role of inflammation is widely discussed in dilated cardiomyopathy, but there are few studies discussing the role of myocarditis in the progression and de-compensation in patients with HCM (Frustaci et al., 2007). We suggest that myocarditis should always be taken into consideration in cases of unexplained deterioration of cardiomyopathy. The mixed phenotype of cardiomyopathy makes the search for more than one cause, especially relevant.

Here, we present a case of progressive mixed hypertrophic and dilated cardiomyopathy in a young patient with a rare genetic cause: bi-allelic mutations in the *MyBPC3* gene.

## Case Description

A 27-year-old man was admitted to the cardiology department with symptoms of biventricular heart failure (NYHA class III), excessive sweating, and pain in the postoperative wound area at the site of thrombectomy 6 months prior to hospitalization.

His family history was unremarkable. Parents were not consanguineous. The father died at the age of 35, in a traumatic accident, and to the best of the patient’s knowledge, had no cardiac complaints. At the time of the first hospitalization, the mother was 56 years old and had no complaints. The proband had a sister who died at 15 days of age due to a congenital heart disease (transposition of the great arteries). No other relative was diagnosed with cardiomyopathy of other cardiac disease before 50 years of age. External risk factors: smoking average of three cigarettes/day for 7 years. No alcohol abuse or other toxic factors were mentioned.

**Figure 1:**
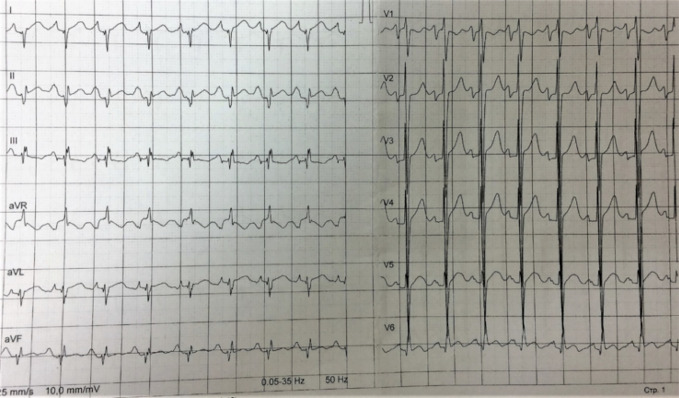
The main milestones of the proband’s medical history. Reconstructed by the patient’s reports and medical records.

Personal medical history with main milestones and medications taken (when possible) was reconstructed from the available medical records (**Figure 1**).

The patient had an average height (174 cm) and weight of 79 kg for his age, BMI 26.1 kg/m^2^, and normal arterial blood pressure (120/80 mmHg). Edema of the leg and slight serous discharge from the wound in the right groin area were found on the initial physical examination.

## Diagnostic Assessment

### Headings Instrumental Investigations (ECG, EchoCG, Cardiac CT, and MRI With Gadolinium Enhancement)

**Figure 2:**
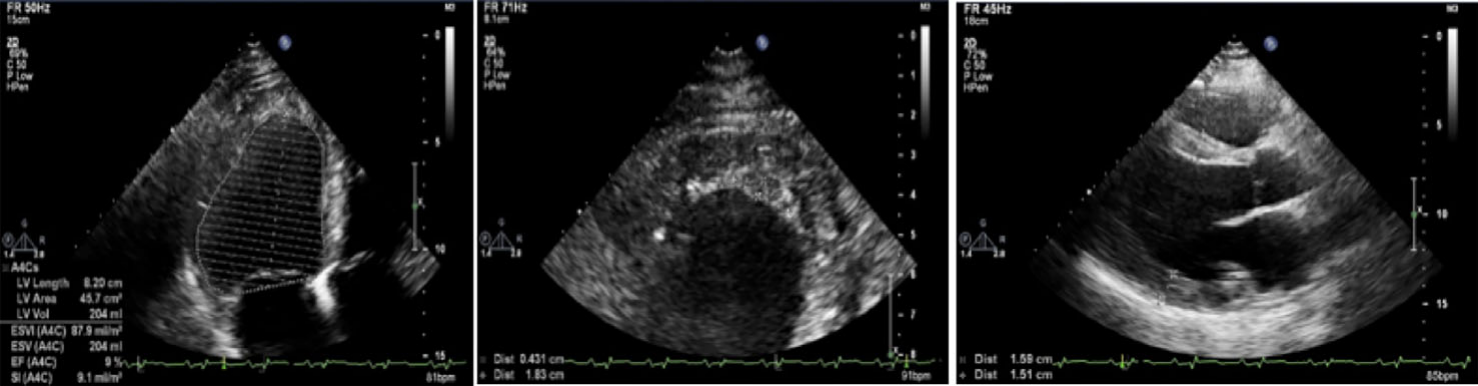
Resting ECG. HR 98 bpm, sinus tachycardia, signs of hypertrophy of both atria and ventricles, and decreased votage of the R waves in standard leads.

**Figure 3:**
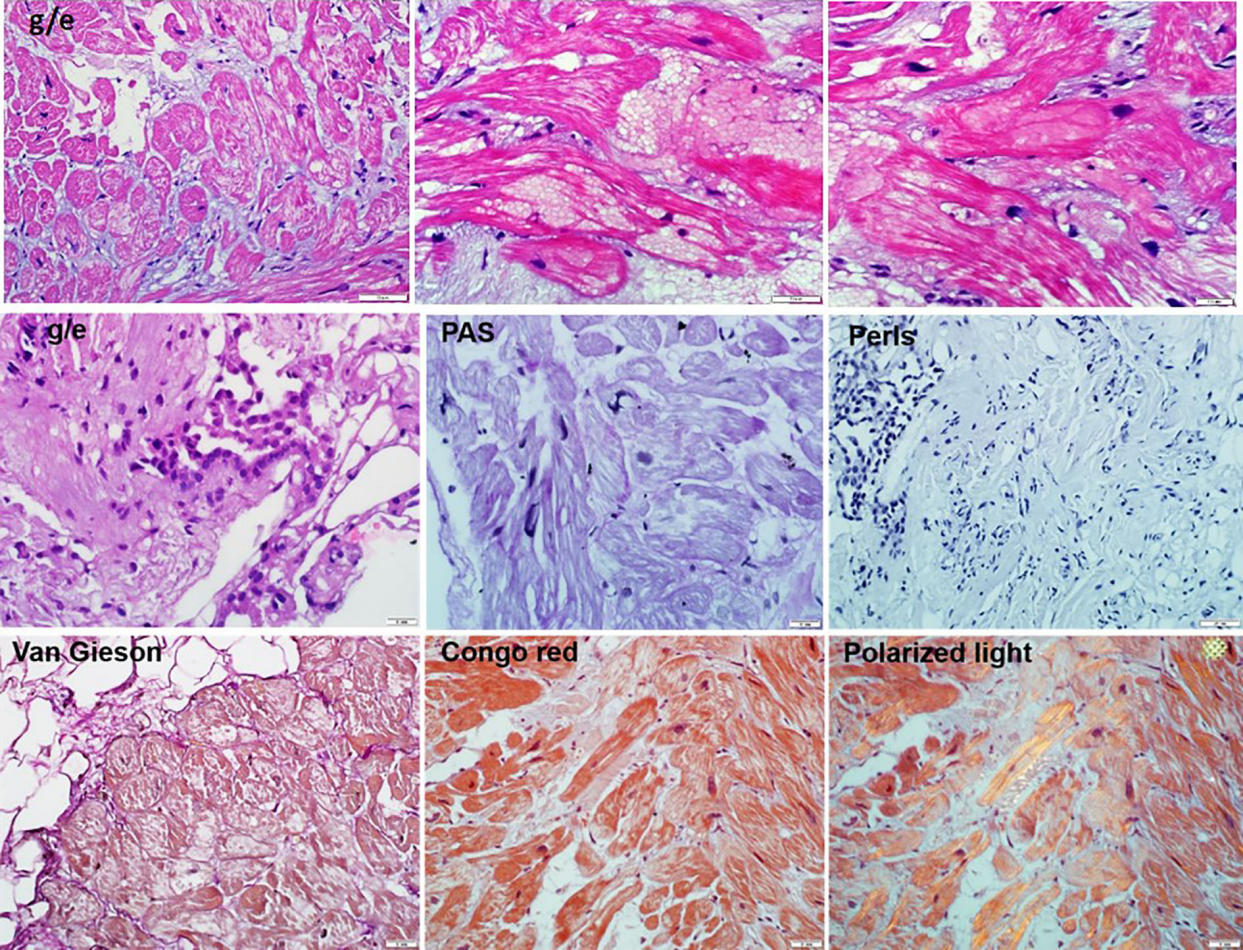
Eсhocardiography. On the left—a severe decrease in left ventricular EF (at this measurement of 9%); in the center—a lining clot in the apex of the left ventricle with dimensions of 4 mm × 18 mm; on the right—hypertrophy of the left ventricular wall to 15–16 mm.

Resting ECG showed HR 98 bpm, sinus tachycardia, signs of hypertrophy of both atria and ventricles, and low R waves in standard leads (**Figure 2**). Holter 24-h ECG monitoring revealed 289 PVBs of two morphologies and two episodes of non-sustained ventricular tachycardia of 4–7 beats 104–227 bpm. Echocardiography had shown (**Figure 3**) enlarged LA (110 ml) and RA (69 ml), dilated LV (end-diastolic diameter 6.4 cm, end-diastolic volume 236 ml, end-systolic volume 192 ml) with decreased contractility (LV EF 20%), and an enlarged RV (end-diastolic diameter 3.1 cm). LV diastolic function was significantly impaired (E, 35 cm/s; A, 23 cm/s; E/A, 1.5; DecT, 70 ms; Emed, 2.2 cm/s; E/Emed 15.8, 5 cm/s). Diffuse cardiac hypertrophy was revealed (IVS 14–16 mm, LV posterior wall 15–16 mm, RV walls 7–11 mm), with LV myocardial mass at 554.84 g. In the region of the apex, a fixed parietal thrombus was detected. Mild mitral, tricuspid, and pulmonary regurgitation were noted. The systolic pressure in the pulmonary artery was 36 mmHg.

Cardiac CT revealed dilation of the LV chamber (69 mm) with a parietal clot spreading from the middle segment of the anterior wall to the apex (5 mm × 19 mm × 90 mm), and LV myocardial hypertrophy (up to 16 mm). In the delayed phase of LV myocardial contrast, it was diffusely heterogeneous. No definite late contrast enhancement or significant coronary stenosis was detected.

On cardiac MRI, the LV was spherical and severely dilated (EDD 71 mm, EDV 120 ml/m^2^) (**Supplementary Figure 1**). The wall thickness of the LV was 14 mm. Increased trabeculation of the LV was found but did not meet the criteria of the LVNC. A decrease in LV contractility with LV EF of 21% was confirmed. Several LGE sites were identified: transmural along the lower wall, subendocardial (up to 60%) in the apex of LV and apical segment of the anterior septum, and extended intra-ventricular LGE (“strip”) in the middle and apical segments of the septum. The apex of the LV was lined with a flat linear thrombus of 3.5 mm across it.

### Laboratory Blood Tests

Initial blood tests revealed erythrocytosis (RBC, 6.85 mcL) and high white blood cell count (WBC, 27.8 × 10U3/ul). Platelet count was normal. Clinical chemistry showed a high hemoglobin level (193 g/L), increased hematocrit (65.8%), elevated erythropoietin (81.3 U/ml), slightly elevated creatinine (119.5 μmol/L), fibrinogen 4.67 g/L, and INR 5.85.

High levels of anti-heart antibodies against cardiomyocytes’ nuclear antigens, endothelial antigens, cardiomyocyte antigens, conduction system fibers, and smooth muscle antigens were detected. No viral genome was detected by virus-specific PCR in the blood.

### Endomyocardial and Bone Marrow Biopsy

**Figure 4:**
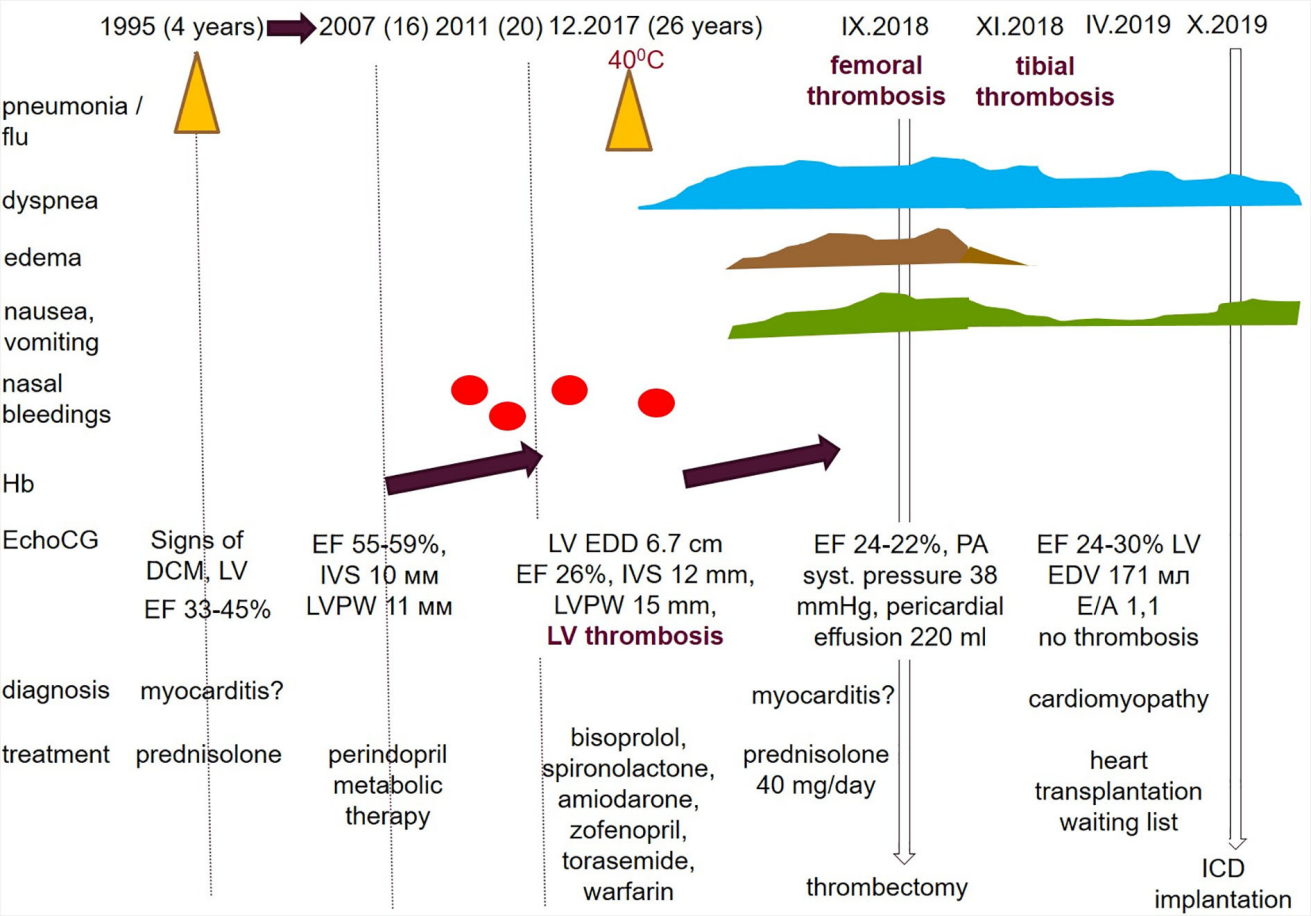
The endomyocardial biopsy of the right ventricle (10–50 micron scale). Hematoxylin eosin staining showed: the endocardium is thin. Cardiomyocytes with foci of enlightenment in the perinuclear zone, disarray, with homogenization of the cytoplasm. In individual cardiomyocytes, there are foci of myolysis with the formation of voids in the cytoplasm. Microvessels with red blood cell sludge phenomenon, sclerosed walls, proliferation of endothelial cells, stenosis of the lumen and single perivascular lymphohistiocytic cells. There are minor hemorrhages, mild sclerosis. Staining of congo red (in non-polarized and polarized light), Perls reaction, the PAS reaction are a negative.

A right ventricular endomyocardial biopsy was performed to verify the diagnosis of myocarditis. A combination of pathologically altered cardiomyocytes, productive vasculitis with thrombosis of individual vessels, and small perivascular lymphocytic-macrophage infiltrates (less than 14 cells) was detected (**Figure 4**). Morphological changes were specific for primary cardiomyopathies in combination with disseminated intravascular coagulation and secondary inflammatory reactions. The genomes of herpes virus, adenovirus, and parvovirus B19 were not detected by PCR in the myocardial biopsy sample.

A bone marrow trepanobiopsy was performed to clarify the origin of erythrocytosis, but no evidence of myeloproliferative disease was found.

### Genetic Analysis

Whole exome sequencing (WES) for the proband’s DNA was performed using a TruSeq Exome library preparation kit (IDT-Illumina) followed by next-generation sequencing on an Illumina system. Reads were aligned to the human genome build GRCh37/UCSC hg19 and analyzed for sequence variants using a custom-developed bioinformatics pipeline. The proband’s WES identified the homozygous deletion chr11:g.47357432_47357458del (ENST00000545968.1: c.2711_2737del) in the *MyBPC3* gene p.2711_2737del. This variant was confirmed in the proband’s DNA in the homozygous state, and in his mother’s DNA in a heterozygous state. In-frame 27 bp deletion leads to the shortening of cardiac myosin binding protein for nine amino acids in the fibronectin III 2 (C7) domain (**Supplementary Figure 2**). This variant was classified as likely pathogenic (Class IV) based on the ACMG (2015) criteria (Richards et al., 2015). No additional pathogenic, likely pathogenic variants nor unique variant of unknown clinical significance was found in the genes responsible for cardiomyopathies or any other storage disorders. Thus, we suggest that bi-allelic deletion in the MyBPC3 gene might be sufficient explanation of the progressive cardiomyopathy in this patient. But, presence of additional variant in the less studied genomic area cannot be completely excluded. Strictly speaking there is an alternative explanation of the NGS and Sanger results for this patient. In theory, it might be a combination of hemizygous deletion due to the whole/partial deletion of the paternal allele (inherited or *de novo*). But, we have no any prove for it and for the best of our knowledge proband’s father had no complaints until he died in car accident at his 35.

No known pathogenic mutations or rare variants were found in the *EPOR* or other genes responsible for the familial erythrocytosis. No unique or known variants with proven clinical significance were found in the genes associated with inherited thrombophilia.

The combination of data from complex evaluation results in the following diagnosis: 1. Familial sarcomeric cardiomyopathy (homozygous deletion p.2711_2737del in the MyBPC3 gene), mixed phenotype (diffuse hypertrophic, dilated). 2. Ischemic cardiomyopathy due to impaired microcirculation with secondary inflammation. 3. Erythrocytosis of unknown origin

Supportive therapy was prescribed: prednisolone 5 mg, bisoprolol 2.5 mg, amiodarone 200 mg, perindopril 2.5 mg, furosemide 40 mg, spironolactone 50 mg, warfarin, acetylsalicylic acid 100 mg, and omeprazole 20 mg. The patient’s condition remains relatively stable for 6 months of follow-up but transient edema, palpitations, sweating, dyspnea at the level of NYHA class 2–3 persisted. Hemoglobin levels returned to normal, but LV dysfunction (LV EF 19%) remained after a temporary improvement (LV EF 28%).

Six months later, the patient experienced another acute thrombosis of the right posterior tibial artery without flotation, and conservative treatment was administered. Due to the development of thyrotoxicosis, amiodarone was replaced with sotalol. Therapy with prednisolone 5 mg per day was continued. In the next 6 months, ICD was implanted. No appropriate shocks were noted. The patient was included in the waiting list for heart transplantation. Unfortunately, despite the normalization of erythrocyte levels and two-component antithrombotic therapy, no improvement in myocardial contractility was noted. Prolonged therapy with prednisolone (at an initial dose of 40 mg per day) and standard cardiotropic therapy had no effect. Heart transplantation appears to be the optimal treatment option.

## Discussion

This clinical case was unusual and challenging in many respects. The combination of severe systolic dysfunction with dilatation of all heart chambers and evident diffuse myocardial hypertrophy is a rare clinical observation. Dilatation of the spherical changed LV with a markedly reduced EF can be a manifestation of both primary DCM and severe myocarditis. At the same time, dilated cardiomyopathy might represent the decompensating stage of any cardiomyopathy.

The wall thickness of the LV met the criteria for HCM. Typical cardiomyocyte disarray found in myocardial biopsy samples was a particular feature of the primary HCM, and the patient had no obvious reason for pressure overload. However, the anatomical variant of cardiac remodeling with diffused, generalized LV walls and right ventricular involvement is more typical for phenocopies of HCM, especially for storage disorders. Among the storage diseases, the earliest decompensation is typical of Danon disease. Therefore, in one of the last studies, unfavorable outcomes (death/transplantation) were noted in one-third of men, with an average age of 21 years (Brambatti et al., 2019). However, the degree of myocardial hypertrophy in our patient was unusually low for Danon disease in men. In addition, he had no typical systemic manifestations of Danon disease, as well as other storage diseases involving the heart (Fabry’s disease, for example). No data was obtained for storage disease in myocardial biopsy after special staining (periodic Acid-Schiff and Congo red in polarized light). Finally, no pathogenic/likely pathogenic genetic variant was found in the genes responsible for the known storage disorders after whole-exome sequencing.

Prolonged undulating course of the disease from 4 years with periods of persistent improvement, possible effects of steroid therapy in the past, the association of rapid deterioration at 26 years with influenza, LV apex thrombosis, preservation of elevated titers of anti-heart antibodies, and mild signs of inflammation in myocardial biopsy despite steroid therapy, might provide evidence of myocarditis. Steroid therapy was started before the patient came to the clinic, which made it difficult to interpret the biopsy data. However, no clinical effect of the steroid prescription was observed despite the absence of viruses in the myocardium.

Inefficiency of steroids and early debut of the disease may have been evidence of primary DCM. However, there was no explanation for diffuse myocardial hypertrophy. The presence of increased trabeculation of the myocardium according to MRI is more typical for sarcomeric cardiomyopathy. Mixed and severe phenotypes have also been reported. Some authors have even proposed the concept of a continuum of sarcomeric cardiomyopathies (Baldi et al., 2010). The cause of rapid decompensation in a previously stable course (myocardial embolic infarct? myocarditis)? was unclear in this case. The pattern of LGE according to MRI could reflect both inflammation and myocardial necrosis. In addition, there were signs of fibrosis, typical of primary myocardial hypertrophy. The diagnosis of isolated sarcomeric HCM was inconsistent with the early manifestation of the DCM phenotype.

According to a large multicenter cohort study, the average age of decompensation for patients with sarcomeric HCM was 45 years (Biagini et al., 2014). The earliest (41 years on average) appearance of final stage of HCM developed in patients with mutations in gene MYH7 and multiple mutations. However, only adult patients were included in the study. According to the data from the Mayo Clinic, out of 2,073 patients with HCM, only eight had EF below 50%, (Killu et al., 2018). Their average age was 44 years. Only five patients underwent LVAD implantation and cardiac heart transplantation 15 years prior to HCM diagnosis. All this data indicated that our case was atypical for an isolated sarcomeric HCM.

The most likely, therefore, was the primary (genetically determined) nature of cardiomyopathy in our patient. One could think of a combination of two or more mutations in the genes of interest for HCM, and we found a bi-allelic mutation in the *MyBPC3* gene. Deletion of nine amino acids raised in the highly conservative C7 FnIII domain (**Supplementary Figure 2**) framed with amino acids from 872 to 967 (Karsai et al., 2011). Several missense mutations (for example, p.Pro873Leu, p. Pro873His, p.Asn948Thr, p. Thr957Ser, p.Thr958Ile) leading to HCM, DCM and LV non-compaction were described in this region (Daehmlow et al., 2002; Nanni et al., 2003; Lekanne Deprez et al., 2006; Ehlermann et al., 2008; Probst et al., 2011). It’s remarkable that most of them were discussed in patients with rapid progression of HCM to a dilated phase even in mono-allelic state (Daehmlow et al., 2002; Nanni et al., 2003; Lekanne Deprez et al., 2006; Ehlermann et al., 2008; Probst et al., 2011). There is no large randomized study regarding the natural course of HCM in patients carrying more than one pathogenic or likely pathogenic allele. However, many particular clinical cases, case-control and observational studies have pointed out that the role of a second genetic hit can be crucial in the age of HCM manifestation (Wessels et al., 2015; Dzemeshkevich et al., 2018; Kissopoulou et al., 2018). Prospective genetic counseling was also challenging. Despite well-accepted autosomal dominant nature of HCM and severe phenotype in our patient, the prognosis for off-springs seems to be favorable. We based it on the fact that his parents were asymptomatic (mother, confirmed carrier, is asymptomatic at 57 y.o.; and father, presumed carrier, was asymptomatic at 35 y.o. when died in accident). Thus, we hypothesize that this deletion p.2711_2737del in mono-allelic state is highly tolerated. But, in the absence of direct confirmation of the health status and genotype of the father, we cannot claim it confidently.

The role of associated myocarditis in the development of rapid decompensation cannot be excluded. However, a mild inflammatory reaction detected in the biopsy may have developed secondary to primary myocardial damage. In any case, there were no indications for aggressive treatment of inflammation.

Finally, it was necessary to exclude *polycythemia vera* as a cause of erythrocytosis, which can aggravate myocardial dysfunction due to microcirculation disorders. Even cases of massive myocardial infarcts in patients with polycythemia and normal coronary arteries are described (Nahler et al., 2017). Myocardial biopsy data confirmed this mechanism to be dysfunctional. There were no strong data to support primary erythrocytosis. No mutations in the genes of interest were identified based on wide genetic screening, and no signs of myeloproliferative disease in bone marrow biopsy were found. Elevated erythropoietin levels could indicate a compensatory mechanism of erythrocytosis due to hypoxia.

Among epigenetic mechanisms, decompensation may influence a lot of the clinical appearance and disease progression. However, we believe chronic microcirculation disorder (sludge, microthrombosis, hemorrhage with cardiomyocyte death) and secondary inflammatory response are responsible for the myocardial damage. It is also impossible to rule out embolic infarction of the lower wall of the left ventricle or necrosis due to intravascular clogging.

Correct diagnosis influence a lot the treatment strategy, long-term prognosis, life style modifications, and reproductive strategy. Information about primary nature of disease is usually stressful what raises many questions regarding risk of transmission and health perspectives and may affect quality of life. Communicating the genetic risk information to maximize understanding and promote health is increasingly important given the rapidly expanding availability and capabilities of genomic and reproductive technologies (Lautenbach et al., 2013). In this clinical case, the prognosis for offspring is favorable provided that second partner in the couple has no HCM. This information was very important for our patient. Currently, there are no options of intervention in primary genetic cardiomyopathy. But, currently, several gene therapy approaches have been developed to rescue genetic defects in “sarcomeric” genes, and, especially, in the *MyBPC3* (Prondzynski et al., 2019). We believe that accumulation of information about natural course of HCM caused by particular mutations and their combinations may provide important insights of new treatment strategies.

## Conclusion

Here, we present a rare case of mixed hypertrophic and dilated cardiomyopathy in a young patient complicated by myocarditis, erythrocytosis, and recurrent thrombi. Disease progression was more severe than usual in classic HCM patients, but relatively long lasting for pediatric DCM first diagnosed at 4 years. Etiological diagnosis was challenging. It required a differential diagnosis between primary, inflammatory, and ischemic myocardial damage. Whole exome sequencing revealed bi-allelic deletion in the *MyBPC3* gene, but erythrocytosis remained unexplained. This observation supports the hypothesis that the co-existence of the genetic causes and additional external factors (thrombosis, inflammation, etc.) contribute significantly to the phenotype and HF progression, and it is important to reveal all of them. Successful diagnostic search and optimal management is only possible with a multidisciplinary team and a whole spectrum of investigations from cardiac imaging and myocardial biopsy to deep genotyping. Experimental research including iPS-CMs studies are needed for the detailed evaluation of the effect of the p.2711_2737del variant in hetero- and homozygous state on myosin-binding protein C3 functioning, and for further insight on molecular mechanisms of cardiomyopathy.

### Patient Perspective

“I have many concerns regarding my future. I’ve thought about heart transplantation and came to the referred Centre. I need to live close to this Centre but I don’t have any opportunity to live in Moscow continiously. I would get on the waiting list but have many concerns about risks related to the operation by itself and subsequent immunosupression. So the question is still open. Well, how to say, I will live as long as I can, and how long it left. I think it will get worse, and I sometimes feel it, but I’m not telling anyone in my city what the condition is now, not to relatives nor my doctor from my city. I don’t think it makes sense if no one can really change the course of genetic disease, right?”

## Data Availability Statement

The raw clinical and genetic data supporting this article cannot be placed in public repository due to ethical reason (personal data protection) but it will be available by the corresponding author (ZE, zhelene@mail.ru) upon reasonable request.

## Ethics Statement

Informed written consent was obtained from the patient for this clinical case publication, and all data and figures included in this article.

## Author Contributions

OB, IA, AZ, and SC participated in the diagnosis and treatment of the patient. EK and VS performed histopathological and instrumental investigations. YS and IK performed wet and bioinformatic genetic investigation. EZ performed genetic counseling of the family. OB and ZE prepared the draft of the manuscript. All authors contributed to the article and approved the submitted version.

## Funding

This work was funded by Russian Science Foundation (Grant № 16-15-10421).

## Conflict of Interest

The authors declare that the research was conducted in the absence of any commercial or financial relationships that could be construed as a potential conflict of interest.

## Supplementary Material

Supplementary Figure 1 Cardiac MRI with gadolinium enchamcement. LV was spherical, severely dilated (EDD 71 mm, EDV 120 ml/m^2^), with increased thickness (14 mm) and trabeculae. LV EF of 21%. Several LGE sites: transmural along the lower wall, subendocardial (up to 60%) in the apex of LV and apical segment of the anterior septum, and extended intra-ventricular LGE (“strip”) in the middle and apical segments of the septum. The apex of the LV was lined with a flat linear thrombus of 3.5 mm across it.

Supplementary Figure 2 Schematic representation of deletion of the 27 bp **(**chr11:g.47357432_47357458del (ENST00000545968.1: c.2711_2737del) in the *MyBPC3* gene found in proband in homozygous state at the DNA, mRNA, and protein level.

## References

[B1] Authors/Task Force members Elliott P. M. Anastasakis A. Borger M. A. Borggrefe M. Cecchi F. (2014). ESC Guidelines on diagnosis and management of hypertrophic cardiomyopathy: the Task Force for the Diagnosis and Management of Hypertrophic Cardiomyopathy of the European Society of Cardiology (ESC). Eur. Heart J. 35 (39), 2733–2779. 10.1093/eurheartj/ehu284 25173338

[B2] Baldi M. Sgalambro A. Nistri S. Girolami F. Baldini K. Fantini S. (2010). Clinica e genetica del ventricolo sinistro non compatto: conferma di un continuum nelle cardiomiopatie [Clinical and genetic features of left ventricular noncompaction: a continuum in cardiomyopathies]. G. Ital. Cardiol. (Rome) 11 (5), 377–385. 20860157

[B3] Biagini E. Olivotto I. Iascone M. Parodi M. I. Girolami F. Frisso G. (2014). Significance of sarcomere gene mutations analysis in the end-stage phase of hypertrophic cardiomyopathy. Am. J. Cardiol. 114 (5), 769–776. 10.1016/j.amjcard.2014.05.065 25037680

[B4] Brambatti M. Caspi O. Maolo A. Koshi E. Greenberg B. Taylor M. (2019). Danon disease: Gender differences in presentation and outcomes. Int. J. Cardiol. 286, 92–98. 10.1016/j.ijcard.2019.01.020 30857840

[B5] Daehmlow S. Erdmann J. Knueppel T. Gille C. Froemmel C. Hummel M. (2002). Novel mutations in sarcomeric protein genes in dilated cardiomyopathy. Biochem. Biophys. Res. Commun. 298 (1), 116–120. 10.1016/s0006-291x(02)02374-4 12379228

[B6] Dzemeshkevich S. Motreva A. Nechepurenko A. Korzh D. Tarasov D. Frolova Y. (2018). [Sudden cardiac death prevention in a patient with diffised generalyzed hypertrophic cardiomyopathy in a patient with two mutations in the MYH7 and MyBPC3 genes]. Clin. Exp. Surg. 3 (6), 78–84. 10.24411/2308-1198-2018-13008

[B7] Ehlermann P. Weichenhan D. Zehelein J. Steen H. Pribe R. Zeller R. (2008). et al. Adverse events in families with hypertrophic or dilated cardiomyopathy and mutations in the MYBPC3 gene. BMC Med. Genet. 9:95. 10.1186/1471-2350-9-95 18957093 PMC2584029

[B8] Frustaci A. Verardo R. Caldarulo M. Acconcia M. C. Russo M. A. Chimeni C. (2007). Myocarditis in hypertrophic cardiomyopathy patients presenting acute clinical deterioration. Eur. Heart J. 28 (6), 733–740. 10.1093/eurheartj/ehl525 17309901

[B9] Karsai A. Kellermayer M. S. Harris S. P. (2011). Mechanical unfolding of cardiac myosin binding protein-C by atomic force microscopy. Biophys. J. 101 (8), 1968–1977. 10.1016/j.bpj.2011.08.030 22004751 PMC3192966

[B10] Killu A. M. Park J. Y. Sara J. D. Hodge D. O. Gersh B. J. Nishimura R. A. (2018). Cardiac resynchronization therapy in patients with end-stage hypertrophic cardiomyopathy. Europace 20 (1), 82–88. 10.1093/europace/euw327 29315424

[B11] Kissopoulou A. Trinks C. Green A. Karlsson J.-E. Jonasson J. Gunnarsson C. (2018). Homozygous missense MYBPC3 Pro873His mutation associated with increased risk for heart failure development in hypertrophic cardiomyopathy. ESC. Heart Fail. 5 (4), 716–723. 10.1002/ehf2.12288 29663722 PMC6073032

[B12] Kuusisto J. (2020). Genetics of hypertrophic cardiomyopathy: what is the next step? Heart 106 (17), 1291–1292. 10.1136/heartjnl-2020-317043 heartjnl-2020-317043. 32546508

[B13] Lautenbach D. M. Christensen K. D. Sparks J. A. Green R. C. (2013). Communicating genetic risk information for common disorders in the era of genomic medicine. Annu. Rev. Genomics Hum. Genet. 14, 491–513. 10.1146/annurev-genom-092010-110722 24003856 PMC3862080

[B14] Lekanne Deprez R. H. Muurling-Vlietman J. J. Hruda J. Baars M. J. H. Wijnaendts L. C. D. Stolte-Dijkstra I. (2006). Two cases of severe neonatal hypertrophic cardiomyopathy caused by compound heterozygous mutations in the MYBPC3 gene. J. Med. Genet. 43 (10), 829–832. 10.1136/jmg.2005.040329 16679492 PMC2563166

[B15] Marian A. J. Braunwald E. (2017). Hypertrophic Cardiomyopathy: Genetics, Pathogenesis, Clinical Manifestations, Diagnosis, and Therapy. Circ. Res. 121 (7), 749–770. 10.1161/CIRCRESAHA.117.311059 28912181 PMC5654557

[B16] Maron B. J. Rowin E. J. Maron M. S. (2018). Global Burden of Hypertrophic Cardiomyopathy. JACC Heart Fail. 6 (5), 376–378. 10.1016/j.jchf.2018.03.004 29724362

[B17] Nahler A. Fuchs D. Reiter C. Kiblböck D. Steinwender C. Lambert T. (2017). Myocardial infarction with proximal occlusion of the left anterior descending coronary artery in a 22-year-old patient with polycythaemia vera. Clin. Med. (Lond). 17 (1), 46–47. 10.7861/clinmedicine.17-1-46 28148580 PMC6297597

[B18] Nanni L. Pieroni M. Chimenti C. Simionati B. Zimbello R. (2003). Hypertrophic cardiomyopathy: two homozygous cases with “typical” hypertrophic cardiomyopathy and three new mutations in cases with progression to dilated cardiomyopathy. Biochem. Biophys. Res. Commun. 309 (2), 391–398. 10.1016/j.bbrc.2003.08.014 12951062

[B19] Probst S. Oechslin E. Schuler P. Greutmann M. Boyé P. Knirsch W. (2011). Sarcomere gene mutations in isolated left ventricular noncompaction cardiomyopathy do not predict clinical phenotype. Circ. Cardiovasc. Genet. 4 (4), 367–374. 10.1161/CIRCGENETICS.110.959270 21551322

[B20] Prondzynski M. Mearini G. Carrier L. (2019). Gene therapy strategies in the treatment of hypertrophic cardiomyopathy. Pflugers Arch. 471 (5), 807–815. 10.1007/s00424-018-2173-5 29971600

[B21] Richards S. Aziz N. Bale S. Bick D. Das S. Gastier-Foster J. (2015). Standards and guidelines for the interpretation of sequence variants: a joint consensus recommendation of the American College of Medical Genetics and Genomics and the Association for Molecular Pathology. Genet. Med. 17 (5), 405–424. 10.1038/gim.2015.30 25741868 PMC4544753

[B22] Wang J. Wang Y. Zou Y. Sun K. Wang Z. Ding H. (2014). Malignant effects of multiple rare variants in sarcomere genes on the prognosis of patients with hypertrophic cardiomyopathy. Eur. J. Heart Fail. 16 (9), 950–957. 10.1002/ejhf.144 25132132

[B23] Wessels M. W. Herkert J. C. Frohn-Mulder I. M. Dalinghaus M. van den Wijngaard A. de Krijger R. R. (2015). Compound heterozygous or homozygous truncating MYBPC3 mutations cause lethal cardiomyopathy with features of noncompaction and septal defects. Eur. J. Hum. Genet. 23 (7), 922–928. 10.1038/ejhg.2014.21116 25335496 PMC4463499

