# Supplementary figures and images for: Mixed Hypertrophic and Dilated Phenotype of Cardiomyopathy in a Patient With Homozygous In-Frame Deletion in the MyBPC3 Gene Treated as Myocarditis for a Long Time
Source: Front Pharmacol. 2020 Sep 25;11:579450. doi: 10.3389/fphar.2020.579450 (PMC7546790; doi:10.3389/fphar.2020.579450)

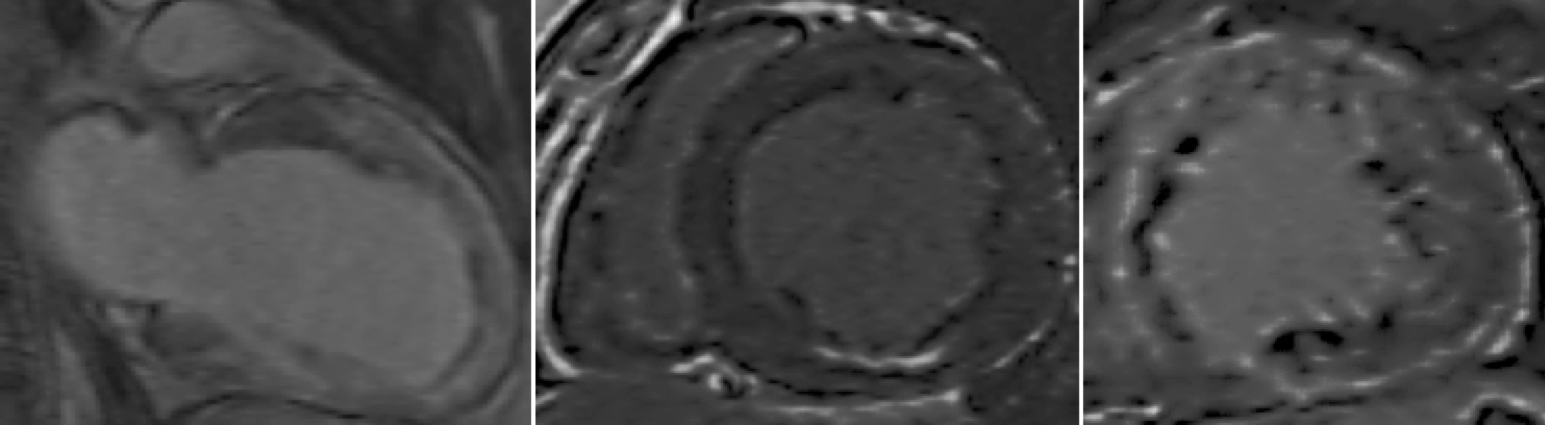

Supplement: Supplementary Figure 1 — Cardiac MRI with gadolinium enchamcement. LV was spherical, severely dilated (EDD 71 mm, EDV 120 ml/m2), with increased thickness (14 mm) and trabeculae. LV EF of 21%. Several LGE sites: transmural along the lower wall, subendocardial (up to 60%) in the apex of LV and apical segment of the anterior septum, and extended intra-ventricular LGE (“strip”) in the middle and apical segments of the septum. The apex of the LV was lined with a flat linear thrombus of 3.5 mm across it. [file Image_1.tiff]

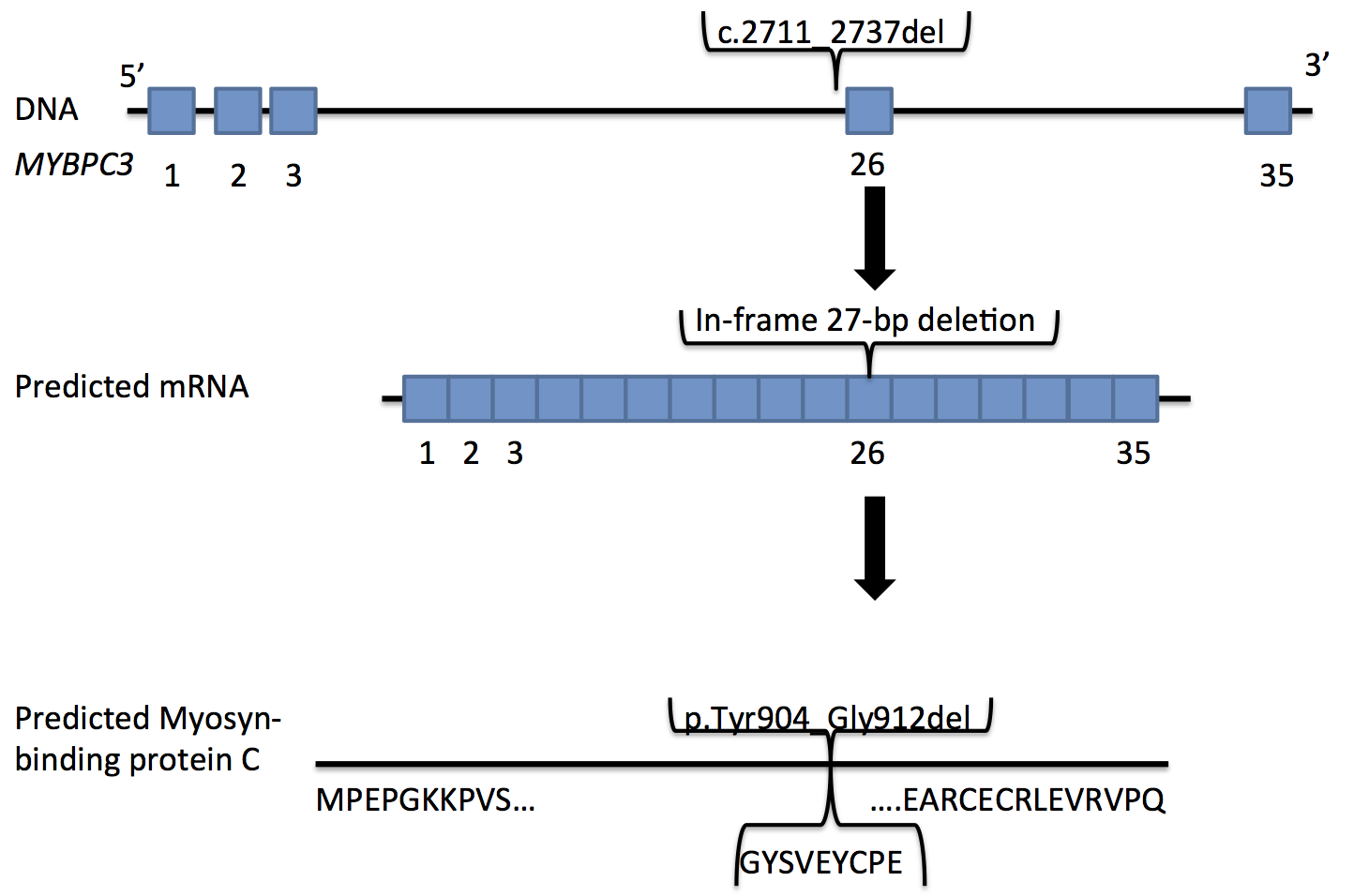

Supplement: Supplementary Figure 2 — Schematic representation of deletion of the 27 bp (chr11:g.47357432_47357458del (ENST00000545968.1: c.2711_2737del) in the MyBPC3 gene found in proband in homozygous state at the DNA, mRNA, and protein level. [file Image_2.tiff]
